# OsPIP2;1 impacts root hydraulic conductance and is a candidate gene for a drought avoidance QTL on rice chromosome 7

**DOI:** 10.1111/plb.70237

**Published:** 2026-06-15

**Authors:** Z. Abubakar, F. S. Khowaja, T. Dasgupta, D. Mieulet, E. Guiderdoni, G. J. Norton, A. H. Price

**Affiliations:** ^1^ School of Biological Sciences University of Aberdeen Aberdeen UK; ^2^ CIRAD, UMR AGAP Institut Montpellier France; ^3^ CIRAD‐INRAE‐Institut Agro University of Montpellier Montpellier France; ^4^ Present address: Department of Biological Sciences Gombe State University Nigeria; ^5^ Present address: 308, 7445 120 Street Delta British Columbia Canada; ^6^ Present address: School of Agriculture and Rural Development Ramakrishna Mission Vivekananda University Kolkata West Bengal India; ^7^ Present address: CIRAD, UMR DIADE Montpellier France

**Keywords:** Aquaporin, drought, hydraulic conductance, Oryza sativa, OsPIP2;1, RNAi

## Abstract

- Mapping drought avoidance qualitative trait loci (QTLs) in the Bala × Azucena population has revealed a locus on chromosome 7 where the Azucena allele increases leaf rolling but reduces leaf drying. A cluster of four aquaporin genes *OsPIP2;1*, *OsPIP2;4*, *OsPIP2;5*, *OsPIP2;9* co‐locate with this QTL. The hypothesis that this gene cluster and the QTL are functionally related was tested, assuming that aquaporin would be linked to drought avoidance via an impact on root hydraulic conductance.
- Genetic differences in these genes was assessed bioinformatically. Physiological assessment of root hydraulic properties was carried out on near‐isogenic lines and RNAi knockdowns of *OsPIP2;1*. For this, a two‐plant pressure chamber was used and a novel technique for assessing root hydraulic flow using osmotic swelling of roots was developed.
- Bala has relatively rare haplotypes for these PIPs that are associated with the aus subgroup of rice, which it shares with the aus cultivar N22 from which it was directly derived in breeding. Allelic variation in root hydraulic flow and conductance was revealed. Notably, the allelic difference in hydraulic flow/conductance was only strongly evident when plants were droughted (e.g. with Azucena allele having an average 70% lower hydraulic flow than the Bala allele). RNAi lines with reduced expression of *OsPIP2;1* had a commensurate reduction in root hydraulic flow and conductance.
- These studies confirm the role of *OsPIP2;1* in root hydraulic flow in rice, and highlight the presence of aus‐specific allelic variation that appears to impact root hydraulic conductance and drought avoidance in rice.

## Graphical abstract

In this study, we identified a rice plasma membrane intrinsic aquaporin (OsPIP2;1) as the candidate gene for a chromosome 7 locus driving drought avoidance, supported by established and novel physiological experiments measuring root hydraulic conductance in near isogenic and RNAi knockdown lines.

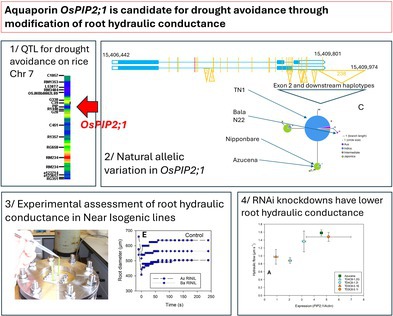

## INTRODUCTION

As with many crops, drought is considered one of the most important abiotic constraints on productivity of rice. For example, almost half of the 52 million hectares of rainfed lowland rice is considered drought prone (Serraj *et al*. 2011). The importance of drought has prompted research on genetic mapping of loci that determine cultivar differences in performance under drought, with these featuring among the first applications of the earliest rice mapping populations (Champoux *et al*. 1995; Yadav *et al*. 1997). The cultivar Azucena is a Philippines landrace with deep roots from the tropical japonica subgroup while Bala is a shallow rooted improved variety bred in India for drought resistance from the indica cultivar TN1 and the well‐studied drought and heat resistant aus cultivar N22 (Price & Tomos 1997). The Bala × Azucena mapping population has been tested for drought in the field in the Philippines and the Cote D'Ivoire (Price *et al*. 2002) revealing several notable loci. The results from that study plus further field drought experiments on the population (in the Philippines (Lafitte *et al*. 2004) and Tamil Nadu, India Gomez *et al*. 2006) formed a part of a meta‐analysis of QTLs in the Bala × Azucena population (Khowaja *et al*. 2009). In that analysis, eight independent drought screens of the population were used to locate some loci of particular note for drought avoidance. Of these, one included a region on chromosome 7 at 50.5 cM, with a 10 cM confidence interval. The physical position of the locus is approximately 15.0 Mbp. A curious feature of this locus, however, that distinguishes it from the other drought avoidance loci detected in this population is the observation that the direction of some trait effects seems to contradict others. This is clearly seen in the Price *et al*. (2002) study where the Azucena allele here increased leaf rolling (indicative of increased drought stress), but decreased leaf drying, probably a better indication of drought‐induced physiological harm (Price *et al*. 2002). This opposition of allelic effects suggests this locus reflects an underlying difference in plant physiology that impacts on the traits measured, where leaf rolling is a mechanism to reduce drought damage by reducing heat gain and water loss while still allowing photosynthesis and was demonstrated to differ substantially between Bala and Azucena (Price *et al*. 1997), while leaf drying is considered as a more simple assessment of drought damage. Yield QTLs were detected here but they are of conflicting direction, such that the Azucena allele increased yield in two drought screens (one in International Rice Research Institute, Philippines and one in Cote D'Ivoire in 1997) but decreased yield in another two (one in same site in Cote D'Ivoire in 1998 and one in Tamil Nadu in India) (Khowaja *et al*. 2009).

The confidence interval revealed by meta‐analysis of QTLs in this region (Khowaja *et al*. 2009) covers 406 genes in the Nipponbare annotated reference (on the Rice Genome Annotation Project, http://rice.plantbiology.msu.edu), including 241 that are not mobile elements (29 annotated as hypothetical and 93 annotated as expressed). Most notable in this region is a cluster of four genes annotated as aquaporins; LOC_Os07g26630, 40, 60 and 90. These four genes are located between 15.35 and 15.41 Mbp on chromosome 7. Three of these have been characterized: *OsPIP2;1* (LOC_Os07g26690), *OsPIP2;4* (LOC_Os07g26630) and *OsPIP2;5* (LOC_Os07g26660) (Sakurai *et al*. 2005). LOC_Os07g26640 is not homologous to the well characterized PIPs of rice (as presented by Sakurai *et al*. 2005), and in MSU annotation, it has no full length cDNA or EST homology suggesting it might be a pseudogene. A recent review of aquaporins (Sun *et al*. 2024) does not mention this gene. However, in the Rice Annotation Project annotation (https://rapdb.dna.affrc.go.jp) (Os07g0448200), there is expression evidence from roots that is labelled *OsPIP2;9* while this gene is not described in literature.

Aquaporins are a ubiquitous and extensively studied family of proteins that form membrane pores, which facilitate the transmembrane movement of water and some other small molecules (Chaumont & Tyerman 2014). Aquaporins have a special function in plants because of the critical role that water plays in the regulation of plant physiology, including the conductivity of the plant and its differentiated tissues to water (Chaumont & Tyerman 2014). Variation in hydraulic conductance has been suggested as a key component in determining adaptation to drought in chickpea (Sivasakthi *et al*. 2017) and mulberry (Reddy *et al*. 2017), and in both cases, aquaporins have been implicated. A meta‐analysis of PIP overexpression studies across species concluded a mixed but generally positive outcome on drought stress response (Ren *et al*. 2021).

The number of aquaporin genes in rice was identified as 33 (with 11 plasma membrane intrinsic proteins (PIPs) by Sakurai *et al*. 2005) although more recently Sun *et al*. (2021) have described 35 genes but still 11 PIPs. It is these PIPs that are thought to be primarily related to plant water relations and are further subdivided into three genes in the PIP1 family (*PIP1;1*, *PIP1;2* and *PIP1;3*) and eight genes in the PIP2 family (*PIP2;1*…*PIP2;8*). The functional role of each of these genes has been studied (Sakurai *et al*. 2005, 2008; Sakurai‐Ishikawa *et al*. 2011). Those studies on the Japanese rice cultivar Akitakomachi reveal that *OsPIP2;1* is heavily expressed in roots and moderately expressed in shoots and is less diurnally regulated than most PIPs (2–3 fold higher in day than night), while *OsPIP2;4* and *OsPIP2;5* are predominantly expressed in roots and are substantially diurnally regulated (28 and 145 fold higher in day than night, respectively) (Sakurai‐Ishikawa *et al*. 2011). Sakurai‐Ishikawa *et al*. (2011) also suggest that, while *OsPIP2;4* and *OsPIP2;5* expression is also regulated by transpiration demand, *OsPIP2;1* is not. Importantly, *OsPIP2;1* is the most expressed PIP in rice roots, with expression levels of *OsPIP2;4* and *OsPIP2;5* very much lower, even at midday (Sakurai‐Ishikawa *et al*. 2011). The same study indicates that in the root both *PIP2;1* and *PIP2;5* are heavily expressed in the endodermis where their influence on whole‐root water movement can be speculated to be greatest. Recently, *OsPIP2;1* was targeted for RNAi downregulation by Ding *et al*. (2019) because it was demonstrated to be highly effective in water transport when expressed in yeast (compared to six other PIPs) and because of previous studies linking it or its orthologue to plant water relations. The authors demonstrated a four‐fold reduction in rice root hydraulic conductance and increased sensitively to 15% PEG‐induced osmotic stress when expression of *PIP2;1* was reduced by 50–70%. Knockout of rice *OsPIP2;4* has been shown to reduce root hydraulic conductance (Aya *et al*. 2026). Complicating studies on aquaporins, however, is the observation that the functional structure of the protein, which is a tetramer that creates a membrane pore, can be formed as heterotetramers where multiple PIPs can be incorporated (including a mix of PIP1s and PIP2s) (Paluch‐Lubawa & Polcyn 2024).

Given the central role of aquaporins in the regulation of water movement in rice, these genes (*OsPIP2;1*, *OsPIP2;4*, *OsPIP2;5* and *OsPIP2;9*) are considered excellent functional positional candidate genes for the drought QTL on chromosome 7 in the Bala × Azucena mapping population. We hypothesize that variation in root hydraulic conductance, controlled by the cluster of PIP genes, is the mechanism behind the drought avoidance QTLs detected on chromosome 7. It could be hypothesized that an allele with lower conductance (if water supply to the shoot was slower than demand) would induce greater leaf rolling but might provide protection against leaf drying if it allowed the provision of water for an extended period during the drought period (i.e., if low root hydraulic conductance promoted a conservative use of soil water resources). An extensive field and greenhouse drought study on approximately 200 aus cultivars that fail to find a link between leaf water potential and leaf rolling, but rather linked the latter to leaf morphology (Cal *et al*. 2019). This suggests the link between the supply of water and leaf rolling in drought is not the only mechanism that could explain differences in leaf rolling between accessions, but root hydraulic conductance was not assessed in that work.

Using available genome sequence and transcriptomic data, near isogenic lines derived from the Bala × Azucena population and novel RNAi lines for *PIP2;1* allowed investigations that aimed to show: (i) there is allelic variation between Bala and Azucena in *OsPIP2;1*, *OsPIP2;4*, *OsPIP2;5* and *PIP2;9* sufficient to support the candidacy of these genes, (ii) that natural allelic variation in this region affects plant water relations and root hydraulic conductance and (iii) that this natural variation can be phenocopied by using RNAi to reduce the expression of *OsPIP2;1*. In order to do that we have developed a simple novel method for assessing hydraulic flow in roots.

## METHODS

### Bioinformatics in *OsPIP2;1*, *OsPIP2;4*, *OsPIP2;5* and *OsPIP2;9*

Azucena and Bala genome sequence is available (data have been deposited in the NCBI Short Read Archive Acc_ID SRX9735769 (Azucena) and SRX128324 (Bala)). The spatio‐temporal display of RiceXPro (Sato *et al*. 2011) was used to reveal the tissue specificity of expression of the PIPs in Nipponbare. Affymetrix array data are available for expression of Azucena and Bala in hydroponic rice roots (Norton *et al*. 2008; NCBI GEO series GSE4471) or in leaves under control and drought (Price *et al*. 2010; NCBI GEO Series GSE24048). Haplotype variation in the 4 genes was examined using RiceVarMapv2.0 (Zhao *et al*. 2015), which contains sequence data for Azucena, Bala and the parents used to breed Bala (TN1 and N22).

In *OsPIP2;1*, the Nipponbare and Azucena sequence but not the Bala sequence has 100% sequence homology to a microRNA osa‐miR1436 identified by Sunkar *et al*. (2008). This site appears missing in Bala because of a 238 bp deletion in the 3′UTR. This indel was surveyed using PCR primers CATCGCGGCGTTCTACCACCAGTA and TGGAAACTTGGAACCCCAGCAGTAGC (which gives 859 or 618 bp products) in the Rice Diversity Panel 1 (Zhao *et al*. 2011) where the deletion has the following frequencies among the subgroups; *Aus* = 90%, *Indica* = 88%, *Tropical Japonica* = 74% and *Temperate Japonica* = 20%.

### Development of recombinant inbred near isogenic lines (RINILs)

In order to generate near isogenic lines, residual heterozygosity in the Bala × Azucena recombinant inbred line (RIL) population was exploited. Since the population was developed from seed collected from F_5_ plants (Price *et al*. 2000), each RIL has an expected 6.25% heterozygosity remaining. Examining the 165 markers used to genotype the population, one of the RILs was selected as it was heterozygous for four markers on chromosome 7 (G338 38.6 cM; C39 42.8 cM; R1440 52.3 cM and G20 54.7 cM), representing a 10.4 Mbp region around the centromere (from 7.1 to 17.5 Mbp). This RIL was also heterozygous for non‐target markers G39, RG139, RM221 and RM6, covering a 24 cM (2.6 Mbp) region of chromosome 2 and single markers G187 on chromosome 8, R79 on chromosome 9 and C223 on chromosome 10. From this line, recombinant inbred near isogenic lines (RINILs) were produced through two phases of selfing to reduce the chance of segregation at non‐target regions. In the first phase, plants were selected as heterozygous for marker RM214 (12.78 Mbp on chromosome 7), seeds collected and sown; and then in the second phase, they were selected with the same marker to be homozygous for Azucena or Bala. A further marker AB0702 (also named ‘PIP’ in genetic maps of Bala × Azucena) was used which targets a 7 bp insertion within the *OsPIP2;1* gene in indica accession Pin Gaew 56 (Malz & Sauter 1999) relative to Nipponbare. The primers were forward 5′ CTTCGCCGTGTTCATGGT 3′ and reverse 5′ CTCCTGCTTGTCGTGTGTGT 3′. The 7 bp size polymorphism was detected with high‐resolution agarose electrophoresis.

### Development of RNAi lines

In order to downregulate *OsPIP2;1* (Os07g26690) in cv. Azucena, two artificial micro‐RNAs (amiRNA) T‐DNA constructs (TD3C8 and TD4C8) were prepared. Using the MicroRNA designer website WMD3, two 21‐bp miRNA sequences were selected on the basis of a unique AAGACACATACATTCTACTAC site in the coding sequence of *OsPIP2;1*, for targeting RNAi of *OsPIP2;1* transcripts. The two identified 21‐mers differed by a single nucleotide change in position 1 while the miRNA* sequences differed from the miRNA sequence by changes of nucleotides in positions 12 and 16, creating mismatches in the amiRNA folded structure that triggers the RNA interference process. These miRNA and miRNA* sequences were used to replace by modification PCR the endogenous miRNA and miRNA* of the rice miRNA precursor osa‐MiR528. The final PCR fragment of the modified precursor with flanking attB1 and attB2 sites was inserted by BP cloning into the attP1 and attP2 sites and placed under the control of the maize ubiquitin promoter and the tnos terminator in the pNW55/pCambia 5300 plasmid (Warthmann *et al*. 2008), according to a procedure similar to that detailed in Koegel *et al*. 2017. The two constructs were mobilized in an EHA 105 *Agrobacterium* strain suspension that was used for co‐culture with Azucena mature seed embryo‐derived calli. Procedures for deriving hygromycin‐resistant cell lines and regenerating primary transformants followed those of Sallaud *et al*. (2003). Copy number of the transgene was assessed using RT‐PCR in the T0 plants.

For each of the TD3C8 and TD4C8, up to 10 T1 seeds of eight independent T0 plants (regenerated from independent co‐cultured calli) were grown in hydroponics and assessed for expression of *PIP2.1* using RT‐PCR with primers CCGCTGGTCGTTTTGTTTC and TACAGGCTAAACACATGAGACATCC using three technical replicates. For both TC3C8 and TD4C8, a pair of plants from the same T1 seed (i.e., coming from same T0 plant) contrasting for expression were grown for T2 seeds (see Supplementary Methods). These were designated TD3C8 5.1/E (low expression), TD3C8 5.1/I (high expression), TD4C8 1.2/G (low expression) and TD4C8 1.2/I (high expression of *PIP2.1*). To clarify the nomenclature, TD stands for the Tapash Dasgupta who designed the constructs and made the transgenics, the number 3 or 4 refers to the 21mer used, the C8 refers to the genotype transformed (Azucena in this case), the number after the – indicates the T0 plant (the callus) it comes from (i.e., the original seed packet) and the letter after the number is individual T1 plant number selected based on RT‐PCR results.

### Hydraulic flow of root pieces

The traditional method of measuring hydraulic conductance using a pressure chamber is very time consuming, so a new rapid method of measuring hydraulic flow was developed. This method uses the rate of osmotic expansion when root pieces swell after they have been osmotically shrunken and then placed in water. Root tips (2 cm) were carefully cut from plants and soil removed. Root thickness was measured using a dissection microscope with an eyepiece graticule and then the cut end was sealed with superglue before being treated with plasmolysis solution, 1 M D‐Sorbitol and 1 mM CaCl_2_, for several minutes. The roots were removed from the solution and root thickness was quickly measured again as time zero. Deionized distilled water was gently added to the samples and the diameter recorded at every 5 s. Hydraulic flow was calculated from the initial slope of the plot of diameter against time, with the diameter at time zero being used to calculate the surface area and volume. To demonstrate that the flow rate into the roots was through aquaporins, 0.5 mM HgCl (as used in Maggio & Joly 1995) was included in all solutions for six replicates, which resulted in a 74% reduction in flow rate (*P* = 0.001).

### Root hydraulic conductance of RINILs using the pressure chamber and osmotic swelling

**Fig. 1:**
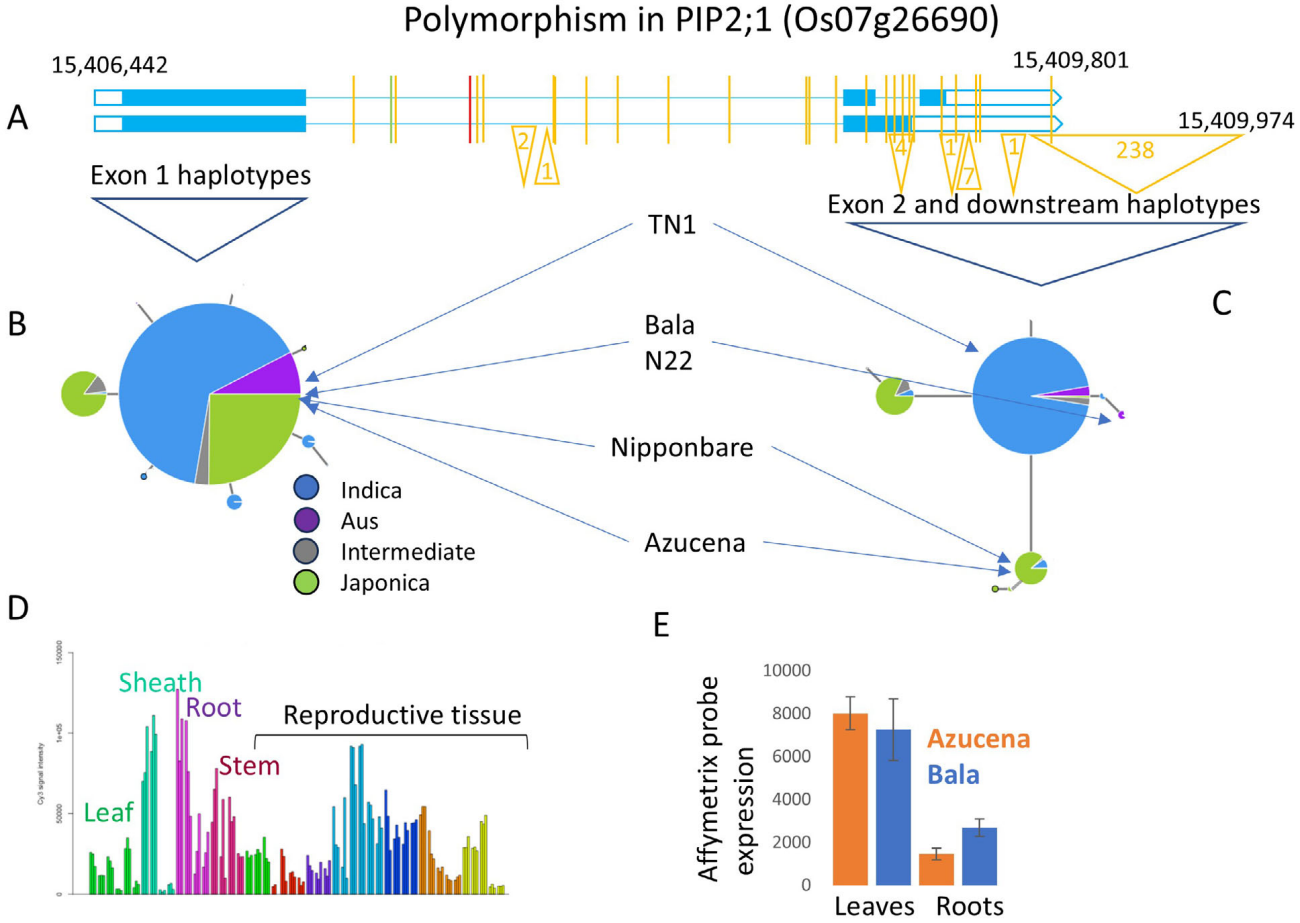
Polymorphism, haplotype diversity and expression in *OsPIP2;1*. (A) The two splice variants of *OsPIP2;1* in Nipponbare based on MSU annotation showing SNPs (vertical lines), insertions (up‐pointing triangles) and deletions (down‐pointing arrows) where red = Azucena, orange = Bala and green is both. Numbers in triangles indicate number of bases. (B) Haplotype network of rice from RiceVarMap2 based on 11 Exon1 SNPs. (C) Haplotype network of rice from RiceVarMap2 based on 26 SNPs from the start of exon 2 to the end of the 238 bp deletion. (D) reproduction of spatio‐temporal expression profile in RiceXPro. (E) Expression of Affymetrix probe Os.11330.1.S1_a_at in field grown leaves and hydroponic roots (see Methods).

**Fig. 2:**
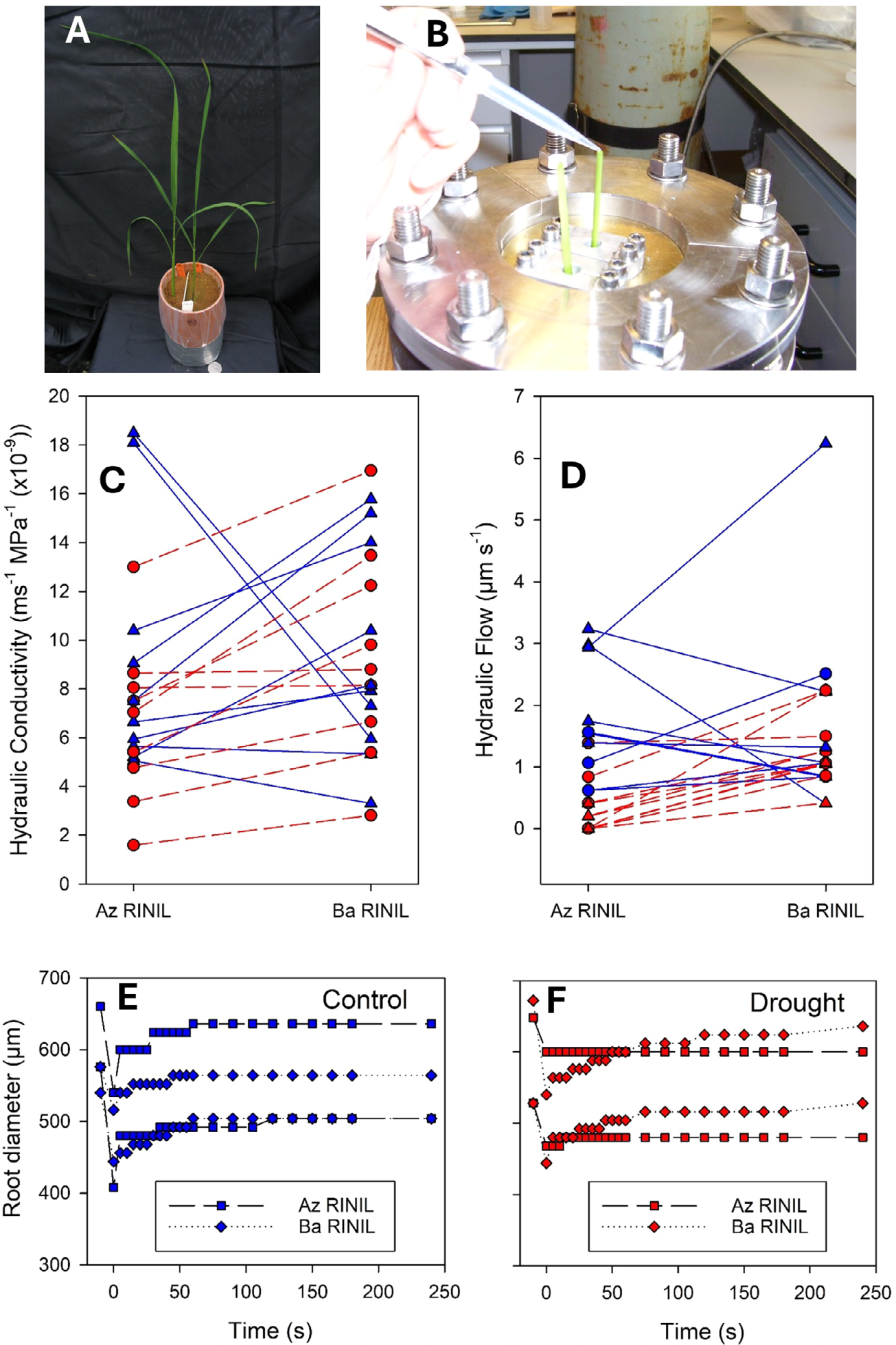
Hydraulic conductance/flow in the roots of near isogenic lines of rice. (A) Photograph of the paired pot system for growing contrasting genotypes; (B) the double‐holed pressure chamber; (C) hydraulic conductance (using pressure chamber) in Azucena (left) and Bala (right) recombinant inbred near isogenic lines (RINILs) under control (solid line) and drought (dashed line) (the paired plants in the same pot are joined by line); (D) hydraulic flow (using osmotic swelling) in Azucena (left) and Bala (right) recombinant inbred near isogenic lines (RINILs) under control (solid line) and drought (dashed line) (the paired plants in the same pot are joined by line); (E) example plots of osmotic swelling of root of well‐watered plants; (F) example plots of osmotic swelling of root of droughted plants.

Experiments were conducted using 18 cm tall, 10 cm diameter tubes filled to a dry bulk density of 1.1 g cm^−1^ with subsoil from an Insch soil association (a sandy loam pH 5.5 as described in MacMillan *et al*. (2006)) that had been air‐dried to 10% gravimetric water content (GWC) and then wetted to 21% gravimetric water content (GWC) using 25 × strength Yoshida's nutrient solution (Yoshida *et al*. 1976) and then sieved to 2 mm. In each pot, two small holes were made 5 cm apart and into each was sown 2 seeds of one or the other RINILs. After germination the seeds in each hole were thinned so that the pot contained one plant of both RINILs 5 cm apart (Fig. 2A). Plants were grown aerobically in a greenhouse, with a day/night temperature of 30/25°C and a relative humidity of ~70% with 12 h supplementary light of 100 μmol m^2^ s^−1^ PAR. For the first 3 weeks, the GWC was maintained at 21% by weighing. After that, half of the pots were subjected to a drought by reducing the GWC to 10% (approximately −600 KPa matric suction in this soil) for a further 3 weeks. It took 6 days to reach the target weights, which were then maintained by twice daily irrigation. Hydraulic conductance was measured simultaneously on both plants in a pot using a specially designed pressure chamber with a two‐holed lid. The plant pot was immersed in water at least 1 h before measurement and excess water was drained from the soil through the bottom of tube. All lateral tillers were cut off below the level of the lid of pressure chamber, leaving only the main stem above the level of the lid. The gap between the main stems and the walls of the lid surrounding the main stem in the holes were tightly sealed using the bungs made of silicon rubber dental impression material (Blend‐a‐med Forschung, Schwalbach, Germany or Affinis Perfect Impressions REF 6610, Colten/Whaldent AG, Switzerland). The hydrostatic pressure in the chamber was raised in steps of 0.2 MPa from 0.3 up to 1.1 MPa above atmospheric. At each pressure, the volume of liquid exuded over a 10 min period was collected at the cut surface of the main stem of both plants simultaneously (Fig. 1B). Transverse sections of the stem taken on several stems after measurements indicated no damage to xylem. After measuring hydraulic flow, roots were washed, separated and root surface area was measured by computerized scanning using an image analysis system (WinRHIZO; Regent Instrument). The gradient of the flow rate to the pressure was used to calculate the hydraulic conductance by dividing by the root surface area. Finally, dry weights of roots were recorded. A strong correlation between root surface area and root dry weight measured on 18 plants (r = 0.867) indicated that in future, root dry weight could be used to assess root surface area using the equation: root surface area (m^2^) = 4.76 × 10^−5^ root dry weight (g) + 0.00217.

Additional experiments using methods described were conducted where instead of the pressure chamber, the root osmotic swelling method was used to assess hydraulic flow.

To test the impact of mild drought, an experiment was conducted using the same conditions as above except the pots had a 9.5 cm diameter (and contained 1.1 l of the same soil at 1.1 g/l dry bulk density) but after 3 weeks well watered, three treatments were applied: 21% (control), 13% (mild drought) and 10% (severe drought) GWC water treatments for 3 weeks. It took 14 and 16 days to gradually reach the 13% and 10% target GWCs (respectively). They were harvested at 8 weeks when relative water content was assessed on the youngest fully expanded leaf while root and shoot length and mass were assessed. Hydraulic flow was assessed osmotically on cut root tips.

### Assessment of *OsPIP2;1* gene expression and hydraulic flow in hydroponically grown RNAi lines

Three day old germinated seedlings were grown in a hydroponic system essentially the same as described in Price *et al*. (1997). Briefly, up to 42 plants were sown into plug trays suspended over 45 l tubs containing ½ strength Yoshida's nutrient solution for the first 2 weeks, then full strength solution for the final week. Nutrient was replaced every week and pH adjusted to 5.5 every day. Plants were grown in the greenhouse with 100 μmol m^2^ s‐1 PAR of supplementary light. At harvest, roots were sampled for osmotic assessment of hydraulic conductance in root pieces as described above and for *OsPIP2;1* gene expression. For expression, RNA was extracted using a Qiagen RNeasy Mini Kit. Quantitative real‐time PCR (qRT‐PCR) was conducted on an Opticon DNA Engine 2 (MJ Research, now Bi‐Rad) using the DyNamo HS SYBER Green qPCR kit (Thermo Scientific). Primers for PIP2.1 were CCGCTGGTCGTTTTGTTTC and TACAGGCTAAACACATGAGACATCC giving a 119 bp product. Expression was expressed relative to the rice actin (RAC1) control using primers of Sakurai *et al*. (2005) TGGTCGTACCACAGGTATTGTGTT and AAGGTCGAGACGAAGGATAGCAT, which gave a 105 bp product. In all qRT‐PCR, there were five biological replicates each with three technical replicates. Expression of each gene was calibrated against qRT‐PCR conducted on serial dilutions of PCR amplicons of known concentration.

### Assessment of hydraulic flow using the pressure chamber in soil‐grown RNAi lines

Pots used were 18 cm tall cylinders filled with 1.225 g dry weight (1.1 g/l) of the same subsoil used previously also saturated with Yoshida's nutrient solution (as described above). In each pot two plants were established, one of both matching pairs of T2 lines generated through the RNAi pipeline (either TD4C8 1.2/G and I or TD3C8 5.1/ E and I) in the greenhouse described above in February and March 2013. These were grown under 21% soil GWC for 3 weeks after which they were subjected to one of three treatments, 21%, 13% or 10% GWC achieved by withholding water essentially as described in 2.6. At 6 weeks of age (at the end of the third week of the drought treatment), the hydraulic conductivity was assessed as described in 2.5 above.

## RESULTS

### Sequence and expression differences between Azucena and Bala

Sequence of Azucena (25×) and Bala (55×) aligned to Nipponbare was examined for the four PIP genes and is summarized in Fig. 1 (for *OsPIP2;1*) and Figs [Link], [Link], [Link] (for *OsPIP2;4, 5 and 9*). The Azucena *OsPIP2;1* allele has two synonymous SNPs relative to Nipponbare in the first intron while Bala has 25 SNPs with Nipponbare plus four small deletions, a 7 bp and a 1 bp insertion and a 238 bp deletion. This large deletion that includes the sequence of microRNA osa‐miR1436 and its presence in Azucena and absence in Bala has been validated using PCR and sequencing PCR products. The deletion removes the last 65 bp of the 3′ UTR (Fig. 1A). However, none of these variations (in Azucena or Bala) changes the predicted protein sequence. Surveying the frequency of the 238 bp deletion in the Rice Diversity Panel 1 revealed it to be dominant in indica, aus and temperate japonica accessions (88, 90 and 73% of accessions, respectively, have the deletion), while it was much rarer in tropical japonicas (only 21%). Haplotype variation using RiceVarMap2 revealed one dominant variant in the first exon, and Azucena and Bala were the same (Fig. 1B). However, analysis of the end of the gene revealed Bala had a rare aus‐dominated haplotype, while Azucena shared a quite different japonica‐dominated haplotype with Nipponbare (Fig. 1C). Bala has the same allele as its N22 parent while its other parent TN1 has a common, indica‐dominated haplotype. Expression data from RiceXPro indicate that *OsPIP2;1* is heavily expressed in most tissues but especially in sheaths and roots (Fig. 1D). Transcriptomics data indicate higher expression in leaves than roots, but do not suggest differences in the expression of *OsPIP2;1* between Azucena and Bala (Fig. 1E).

For *OsPIP2;4*, the sequence of both Azucena and Bala are very different to Nipponbare with more than 40 SNPs including 11 in the exons, six deletions and three insertions (Fig. S1A). Importantly, both Bala and Azucena appear to share a very similar allele for this gene although in the 500 bp immediately upstream of the gene, Bala has several SNPs (including within 100 bp) that are not shared with Azucena suggesting the regulation of expression of this genes might be different between Bala and Azucena. Haplotype analysis of the gene indicate that Bala shares a rare aus‐dominated haplotype with N22, which is slightly different to the japonica‐dominated allele of Azucena (Fig. S1B) that is very different to that of Nipponbare. The expression of this gene is predominantly in roots according to RiceXPro (Fig. S1C) which is confirmed in Bala and Azucena (Figure S1D) which reveals 10× higher expression in Bala, than Azucena.

For *OsPIP2;5* Azucena has 7 SNPs with Nipponbare all of which fall in exons (6 in first exon, 1 in last) and two of which are non‐synonymous and one (aa18 P ➔ Q) causes a change in a conserved region (Fig. S2A). Bala shares only two of these SNPs but has 2 different SNPs in the first intron, a SNP in the 5′UTR and two in the 3′UTR. Importantly, it has a 33 bp deletion in the 5′UTR. Only one polymorphism is non‐synonymous and is the same as Azucena. It is not the non‐synonymous SNP of Azucena that appears to be in a conserved part of the sequence. Azucena has a japonica‐dominated haplotype for this gene similar to Nippobare, which is quite distinct from that of most indicas while Bala and N22 have a rare, aus‐dominate haplotype (Fig. S2B). Like *OsPIP2;4*, this gene appears to be largely root specific as revealed in RiceXPro (Fig. S2C) and array transcriptomics (Fig. S2D) while the later do not suggest differences in the expression of *OsPIP2;5* between Azucena and Bala.

For *OsPIP2;9*, Azucena has 12 SNPs, a 4 bp deletion and two insertions (21 bp and 4 bp) relative to Nipponbare. Bala has all of these polymorphisms except one SNP but has four other SNPs (Fig. S3A). As with the other PIPs, Bala and N22 share a rare aus‐dominant haplotype, while Azucena has one that is similar to the most common and indica‐dominant haplotype that TN1 has and quite different to the Nipponbare haplotype that is also japonica‐dominated (Fig. S3B). The tissue expression pattern of *OsPIP2;9* appears almost identical to *OsPIP2;4* (Fig. S3C), but it is not different between Azucena and Bala (Fig. S3D).

### Root hydraulic conductance of RINILs

The drought treatment significantly (*P* < 0.05) impacted the relative water content only of the Bala allele plants, which reduced to 76.4% ± 3.4% (SE) (cf 84% in controls and Azucena allele droughted). Assessing root hydraulic conductance using a pressure chamber revealed variable results between pots but the trait did not differ between water treatments (Fig. 2C). The genotypes were not different in root surface area in either treatment (data not shown) or in hydraulic conductivity under control conditions. However, in all but two droughted pots, the hydraulic conductivity was higher in the Bala allele than the Azucena allele, and overall, the hydraulic conductivity was significantly (*P* = 0.006) higher (by an average of 42%) in the Bala allele than the Azucena allele in a paired *t*‐test.

When using root swelling to assess hydraulic flow, 1 M sorbitol reduced the diameter of control roots by 18–28% (Fig. 2E). On rehydration, diameter increases rapidly over the course of about 60 s before plateauing. In roots from droughted plants, the magnitude of diameter increase is commonly (but not always) much less than in control plants (Fig. 2F), but the time scale over which the diameter increases appears similar. Using the initial slope of the rapid increase in diameter to measure hydraulic flow revealed variation between genotypes only in the droughted treatment (Fig. 2D). In the control treatments, no genotypic pattern was revealed. In contrast, however, in every droughted pot, the hydraulic flow was higher in the Bala RINIL than the Azucena RINIL, and in 6 out of 12 droughted pots, the hydraulic flow of the Azucena RINIL was zero. Statistically, the hydraulic flow of droughted Azucena RINILs was lower than Bala RINILs (paired *t*‐test *P* < 0.001), with the Bala allele being 70% higher than the Azucena on average.

**Fig. 3:**
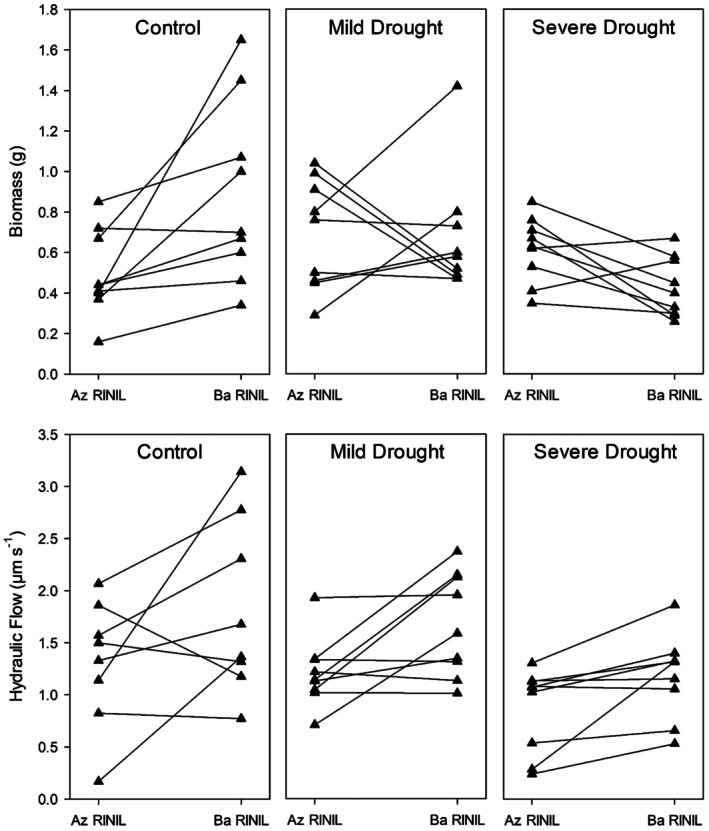
Plant biomass and hydraulic flow (by osmotic swelling) of contrasting recombinant inbred near isogenic lines (RINILs) under three water treatments. Biomass (top) and hydraulic flow as assessed by osmotic swelling (bottom) of contrasting Azucena and Bala in well watered (left), mild (middle) and severe (right) grown in paired pots. The solid line links the two plants in each pot.

In order to confirm the observations made on root hydraulic flow in experiments above, the paired RINIIL approach was applied to an experiment with three levels of water treatment. During the water stress treatments, stomatal conductance was measured on Day 40, 47 and 54. The treatment reduced stomatal conductance, but no difference was detected between the RINILs (data not shown). At harvest, RWC did not differ between RINILs and was only slightly reduced by the severe stress treatment. Plant dry weight was different between RINILs (Fig. 3A), being almost significantly (*P* = 0.077; paired *t*‐test) higher in the Bala RINIL in control conditions and higher (*P* = 0.003) in the Azucena RINIL in the severe stress. These differences in total plant mass were reflected in both the root and shoot dry weight, while root:shoot ratios did not differ between genotypes (or treatments). The treatments reduced hydraulic flow of root pieces (Fig. 3B) (ANOVA *P* = 0.022), with severe drought lower than controls (Tukey's test). Across all treatments, root hydraulic flow was significantly lower in the Azucena RINIL than the Bala RINIL (paired *t*‐test for all data *P* = 0.001) and was lower for the Azucena RINIL in the mild stress alone (by 38%, *P* = 0.029) and the severe drought (by 36%, *P* = 0.020), but the genetic difference (36%) was not significant for the control treatment.

### Gene expression and hydraulic flow in hydroponically grown *OsPIP2;1* RNAi lines

**Fig. 4:**
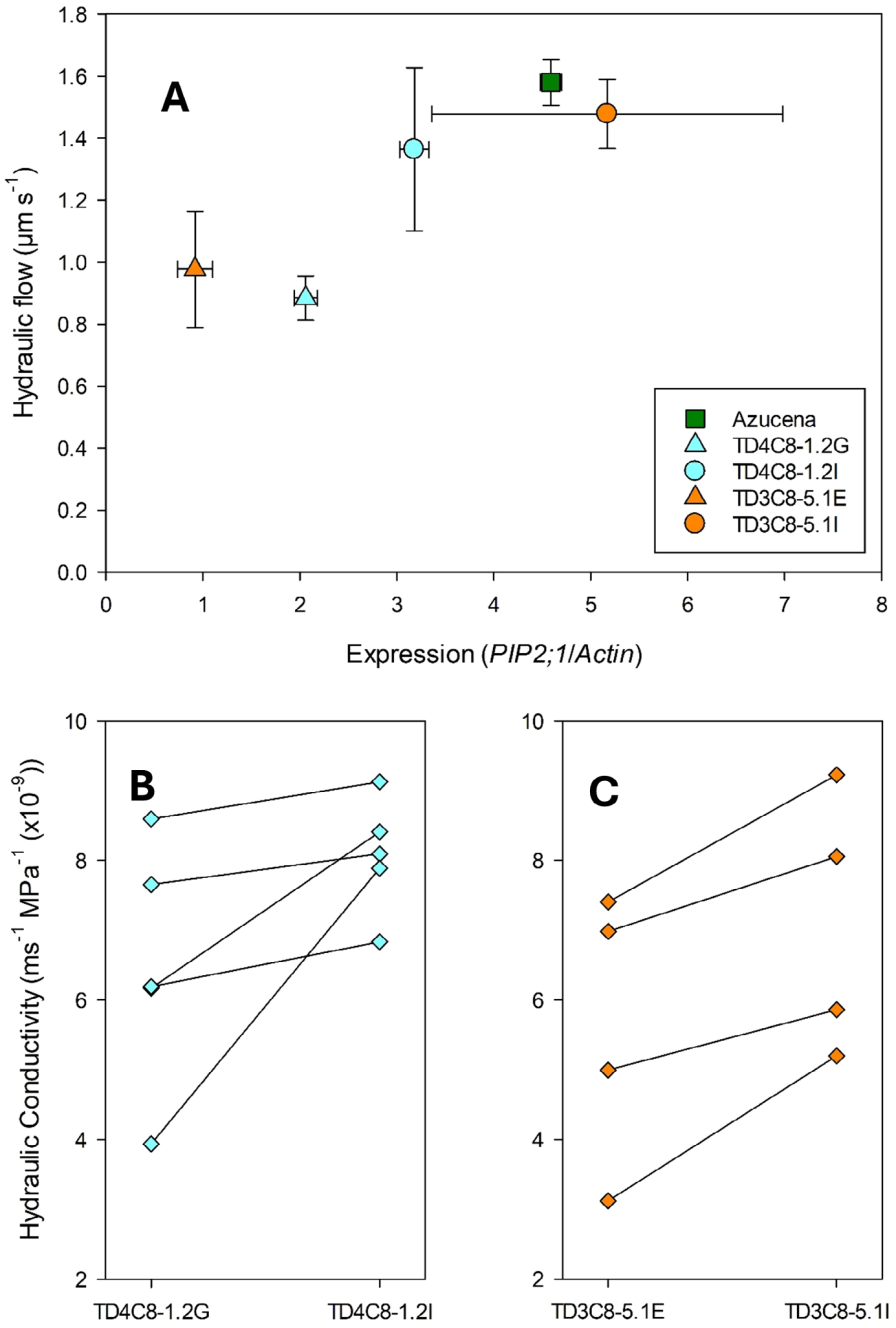
Expression and hydraulic conductance/flow in RNAi lines of *OsPIP2;1*. (A) Plot of the relative gene expression of OsPIP2;1 and hydraulic flow (using osmotic swelling) of four RNAi lines from two target 21mer (TD3 and TD4) and Azucena (the cultivar the lines are developed in) grown in hydroponics. (B, C) Hydraulic conductance (using pressure chamber) of the same lines grown in soil in paired‐plant pots where the contrasting genotype of the same pot are joined by a solid line. Pairs selected within the same transformation to be high *versus* low in expression in hydroponic roots (TD4C8 1.2/I *versus* TD4C8 1.2/G B and TD3C8 5.1/I *versus* TD3C8 5.1/E C).

Because *OsPIPs* on chromosome 7 were candidate genes for the drought QTL detected there and the genetic differences in root hydraulic conductance measured in near isogenic lines (above), efforts were made to produce RNAi knockdown transgenics of *OsPIP2;1* since it is the most highly expressed of the candidate aquaporins. Using quantitative PCR for *OsPIP2;1* on the roots of hydroponically grown plants, two pairs of transgenics either with low or normal *OsPIP2;1* expression from two independent transformations were recovered (Fig. 4A). The same roots were used to measure root hydraulic flow using the osmotic cut root method and revealed strong genotypic differences of about a 40% reduction in flow, which matched the expression pattern of PIP2;1 (Fig. 4A).

### Hydraulic conductance using the pressure chamber in soil‐grown RNAi lines

The same RNAi lines identified in hydroponic above were grown in soil‐filled pots for 6 weeks under well‐watered conditions and subsequently root hydraulic conductance measured with a pressure chamber using the paired genotype approach used for the RINILs. In every pot, the RNAi line with reduced expression had reduced hydraulic flow, with about a 10% reduction in the TD4C8 1.2 pair (Fig. 4B; almost significant *P* = 0.084; paired *t*‐test) and 25% in the TD3C8 5.1 pair (*P* = 0.015) (Fig. 4C). With both pair comparisons combined, the significance of the difference in hydraulic conductance between the high expressing and the low expressing lines was *P* = 0.004 (paired *t*‐test).

## DISCUSSION

A drought avoidance QTL almost invariably detected around 50 cM of chromosome 7 in the Bala × Azucena mapping population (Khowaja *et al*. 2009) was notable for the opposing direction of allelic effects for leaf rolling and leaf drying, where the Azucena allele increased leaf rolling and decreased leaf drying (Price *et al*. 2002). In this genome region, there is a cluster of PIP aquaporins allowing a hypothesis to be formulated that theorizes they are functional candidate genes for the QTL. The logic was, (i) allelic variation in aquaporins in this location altered root hydraulic conductance, (ii) the reduced hydraulic flow of one allele might lead to higher leaf rolling when the plant was under drought stress (because of a greater difference in transpiration demand and root supply of water), (iii) but it would result in reduced leaf drying (which occurs after rolling) because of a more conservative use of water. The first hypothesis is tested in this study. If all hypotheses are true, the direction of allelic effects in leaf rolling and leaf drying traits suggested that the Azucena allele should be the one that is more conservative in water use, and hence, the allele with lower hydraulic conductance, at least under drought.

Examination of Bala and Azucena sequence data suggests there may be important variation between Bala and Azucena in all of four PIPs. For *OsPIP2;1*, it is the presence of a large deletion in the 3′UTR which is considered most noteworthy since there is no evidence of non‐synonymous polymorphism within the coding region of the gene itself. Of particular interest is the fact that the sequence deleted has a recognition site for a microRNA suggesting that there is allelic variation in the way this gene is trans‐regulated. This microRNA (osa‐miR1436) has been linked to heat stress response (Mangrauthia *et al*. 2017). For *OsPIP2;4*, substantial sequence variation upstream seems to be reflected in differences in expression levels in Azucena and Bala, which are easily detected (root expression is 10 lower in Azucena than Bala). For Azucena and Bala, *OsPIP2;5* has several polymorphisms, but no evidence of differences in expression. It is a non‐synonymous SNP in Azucena in a conserved region of the protein not shared by Bala that is considered the most noteworthy. *OsPIP2;9* is considered the least likely causative gene because there is no direct evidence of its function; the sequence polymorphism between Azucena and Bala is limited, and their expression seems the same.

In order to test physiologically the hypothesis of PIP gene candidacy, near isogenic lines were produced using the residual heterozygosity available in the recombinant inbred lines of the Bala × Azucena population. RINILs differing in this region of chromosome 7 were shown to have altered root hydraulic conductance as measured using a pressure chamber, but only when droughted (Fig. 2). There was high variability in hydraulic conductance between replicate pots (as clearly seen in Fig. 2C), which we speculate relates to interactions between the soil and the root. This makes studying root hydraulic conductance in soil‐grown plants difficult. For that reason, it was decided to assess the genetic differences using pairs of genotypes in the same pot, which was facilitated by a pressure chamber with a double opening. Of critical importance for supporting the candidacy of aquaporins for the drought avoidance QTL, the Azucena allele has reduced hydraulic conductance, matching the hypothesis that original linked PIPs to QTLs for leaf rolling and leaf drying under drought in the field.

The pot experiments reported in Fig. 2C do not reveal an impact of drought on root hydraulic conductance but rather a genotype by drought interaction where the Azucena RINIL had lower hydraulic conductance only under drought. Probably because of the difficulty in measurement, there have been limited studies of root hydraulic conductance in rice in response to drought. However, Grondin *et al*. (2016), examined the impact of drought stress on six rice cultivars including Azucena and showed a reduction of root hydraulic conductance by 8–70% by drought treatment. Infusing the soil‐filled pots with azide to inhibit aquaporins demonstrated that the majority of hydraulic flow was through aquaporins. They also measured root expression of eight PIPs including *OsPIP2;1* and *OsPIP2;4*. While expression varied considerably between cultivars, in Azucena *OsPIP2;4* expression was not detected (confirming the array results in Norton *et al*. 2008). In general, PIP expression was decreased by drought. Henry *et al*. (2016) have shown that root hydraulic conductance of rice is lowered by drought and differs between rice cultivars. Cultivar differences in the expression *of OsPIP2;1* and responsiveness to drought have been reported in leaves of mature pot‐grown rice plants (Yooyongwech *et al*. 2013) with cultivar KDML 105 being higher than MT 401 and PT1 under control conditions and with KDML 105 and PT1 reducing very markedly under drought. Together the research links aquaporin expression and function to root (and perhaps also shoot) hydraulic conductance and drought response in rice.

Because measuring hydraulic conductance is time consuming to measure (4 pots in a day in our experiments), a cut root method was developed that used osmotic swelling rate to measure hydraulic flow that was approximately 8 times faster (Fig. 2E,F). The demonstration that adding the aquaporin‐selective blocker mercury reduced the hydraulic flow by 76% is strong evidence that most of the swelling was from water that passed through an aquaporin. Importantly, using this method clearly revealed differences in trait values (Fig. 2D) that agree very strongly (in terms of treatment and genotype effects) with the pressure chamber measures of hydraulic conductance (Fig. 2C). This strongly suggests the root swelling method is a relatively easy measure of root hydraulic properties that should be valuable to the research community especially since it could be speeded up with image capture instrumentation. The authors are not aware of other published reports of using this method.

Because of the importance of the observations suggesting that hydraulic conductance differed between RINILs, but only under droughted conditions, the experiments were repeated again but including a mild as well as a severe drought (Fig. 4B). This experiment confirmed the genetic difference in osmotically measured hydraulic flow that was revealed in both mild and severe drought but not well‐watered conditions. Importantly, the plant growth data of this experiment (Fig. 4A) suggested that reduced hydraulic flow improves growth under severe drought but impairs it under well‐watered conditions.

The experiments above support the overall hypothesis that allelic variation within PIPs located at 15 Mbp on chromosome 7 are functionally related to the drought QTLs. Given the observations of Sakurai‐Ishikawa *et al*. (2011) of the relative importance of *OsPIP2;1* over *OsPIP2;4* and *OsPIP2;5* based on expression, we focused on altering the expression of *OsPIP2;1* using RNAi. We isolated two pairs of transgenics, based on independent transformation events, where one of the pair displayed reduced *OsPIP2;1* expression and the other did not (Fig. 4A). As expected for RNAi, we did not find knockouts, but rather lines with expression reduced by 35% and 82%. Root hydraulic flow was reduced in the lower‐expressing transgenics when grown hydroponically (Fig. 4B,C) and showed a clear linear association with *OsPIP2:1* expression (Fig. 4A). This work confirms that of Ding *et al*. (2019) who demonstrated 50–70% lower expression of Nippionbare RNAi lines of *OsPIP2;1* which had an approximately 80% reduction in root hydraulic conductance. Huang *et al*. (2021) have created a CRISPR knockout of *OsPIP2;1* in Nipponbare. While this did not impact growth under well‐watered conditions, they demonstrated a decrease in stomatal conductance. The authors did not measure hydraulic properties of the root or impose drought.

It has recently been demonstrated that a knockouts of *PIP2*;4 (T‐DNA mutant and CRISPR) reduced hydraulic conductance in rice roots by more than 50% (Aya *et al*. 2026). There are reasonably numerous studies on the overexpression of *OsPIP2;1* in plants. For example, overexpression of *PIP2;1* from Vetiver grass increased transpiration of soybean under PEG‐induced osmotic stress (Hu *et al*. 2016). There are also a few studies on knockout mutants, such as that of Da Ines *et al*. (2010) who showed 20% reduction in rosette water flux in Arabidopsis lacking *AtPIP2;1*, which was likely related to reduced hydraulic conductance. Together the functional studies on *PIP2;1* demonstrate a role in water transport where lower expression reduces hydraulic conductance. Interestingly Chen *et al*. (2022) showed that *OsRINGxf1* confers drought resistance in rice by targeting the degradation of *OsPIP2;1*, which slows water use. Importantly, the results presented here strongly suggest that natural allelic variation in PIP2s, and possibly *OsPIP2;1* specifically, impact root hydraulic conductance and performance under drought.

A complication to a simple interpretation of these results offered above comes from the overexpression and RNAi studies of Sun *et al*. 2021 on *OsPIP2;3* which has a largely root‐based expression pattern. In their hydroponic study, overexpression increased growth in 20% PEG and reduced PEG‐induced damage, while RNAi did the opposite. Interestingly, RNAi increased the rate of water loss from detached leaves from unstressed plants (while overexpression decreased it), demonstrating that altering the expression of single PIP genes can have widespread physiological changes that are currently difficult to explain. Also, determining a relative importance of each and individual PIP is complicated by the way their basic property (water flow) is regulated by expression, cellular trafficking, heterotetramer formation and gating (Chaumont & Tyerman 2014). Further, it has been demonstrated in maize that drought promotes protein complexes dominated by PIP2s in roots but heterotetramers enriched with PIP1s in leaves (Paluch‐Lubawa & Polcyn 2024).

In conclusion, we demonstrate allelic variation in hydraulic conductance between Azucena × Bala near isogenic lines that differ on chromosome 7 making the trait a good physiological candidate for the drought avoidance QTL located there. We demonstrate an osmotic swelling method for assessing root hydraulic flow that is much faster than the pressure chamber normally used. We show that there is allelic variation at the sequence and expression level in four PIP aquaporin under this QTL. Using near isogenic lines we demonstrate allelic variation at this QTL affects root hydraulic flow that is observable only under drought. Furthermore, we demonstrate that modification of the expression of the *OsPIP2:1* using RNAi alters root hydraulic conductance, providing evidence that allelic variation in this gene might be functionally related to the drought avoidance QTL. This possibility should be further studied taking into account the other PIP2 genes in this locus and the interaction of PIP1s and PIP2s in determining hydraulic conductance.

## AUTHOR CONTRIBUTIONS

ZA co‐designed and conducted half of the experiments. FK co‐designed and conducted half of the experiments. TD designed the RNAi constructs and co‐conducted the molecular work on the RNAi lines. DM assisted TD in the creation of the RNAi lines. EG oversaw the RNAi line production including design, seed production and transport of seed. GN co‐designed and supervised experiments, analysed data, produced figures. AP co‐designed and supervised experiments, analysed data, conducted bioinformatics, produced figures and wrote the first draft. All authors contributed to editing.

## Supporting information


**Fig. S1.** Polymorphism, haplotype diversity and expression in *OsPIP2;4*. (A) *OsPIP2;4* in Nipponbare based on MSU annotation showing SNPs (vertical lines), insertions (up‐pointing triangles) and deletions (down‐pointing arrows) where red = Azucena, orange = Bala and green is both. Numbers in triangles indicate number of bases. (B) Haplotype network of rice from RiceVarMap2 based on 53 SNPs. (C) reproduction of spatio‐temporal expression profile in RiceXPro. (D) Expression of Affymetrix probe Os.8118.1.S1_at in field grown leaves and hydroponic roots (see Methods). * Insertion in 3′UTR is difficult to size due to poor sequence read alignment.

**Fig. S2.** Polymorphism, haplotype diversity and expression in *OsPIP2;5*. (A) *OsPIP2;5* in Nipponbare based on MSU annotation showing SNPs (vertical lines) and deletions (down‐pointing arrows) where red = Azucena, orange = Bala and green is both. Numbers in triangles indicate number of bases. * is SNP 15,377,054 indicated as ‘probably damaging’ in RiceVarMap2. (B) Haplotype network of rice from RiceVarMap2 based on 34 SNPs. (C) reproduction of spatio‐temporal expression profile in RiceXPro. (D) Expression of Affymetrix probe Os.31191.2.S1_x_at in field grown leaves and hydroponic roots (see Methods).

**Fig. S3.** Polymorphism, haplotype diversity and expression in *OsPIP2;9*. (A) *OsPIP2;9* in Nipponbare based on MSU annotation showing SNPs (vertical lines), insertions (up‐pointing triangles) and deletions (down‐pointing arrows) where red = Azucena, orange = Bala and green is both. Numbers in triangles indicate number of bases. (B) Haplotype network of rice from RiceVarMap2 based on 22 SNPs. (C) reproduction of spatio‐temporal expression profile in RiceXPro. (D) Expression of Affymetrix probe Os.44151.1.S1_x_at in field grown leaves and hydroponic roots (see Methods).

**Data S1.** Supplementary methods.
