# Supplementary figures and images for: OsPIP2;1 impacts root hydraulic conductance and is a candidate gene for a drought avoidance QTL on rice chromosome 7
Source: Plant Biol (Stuttg). 2026 Jun 15;28(5):1602–13. doi: 10.1111/plb.70237 (PMC13358641; doi:10.1111/plb.70237)

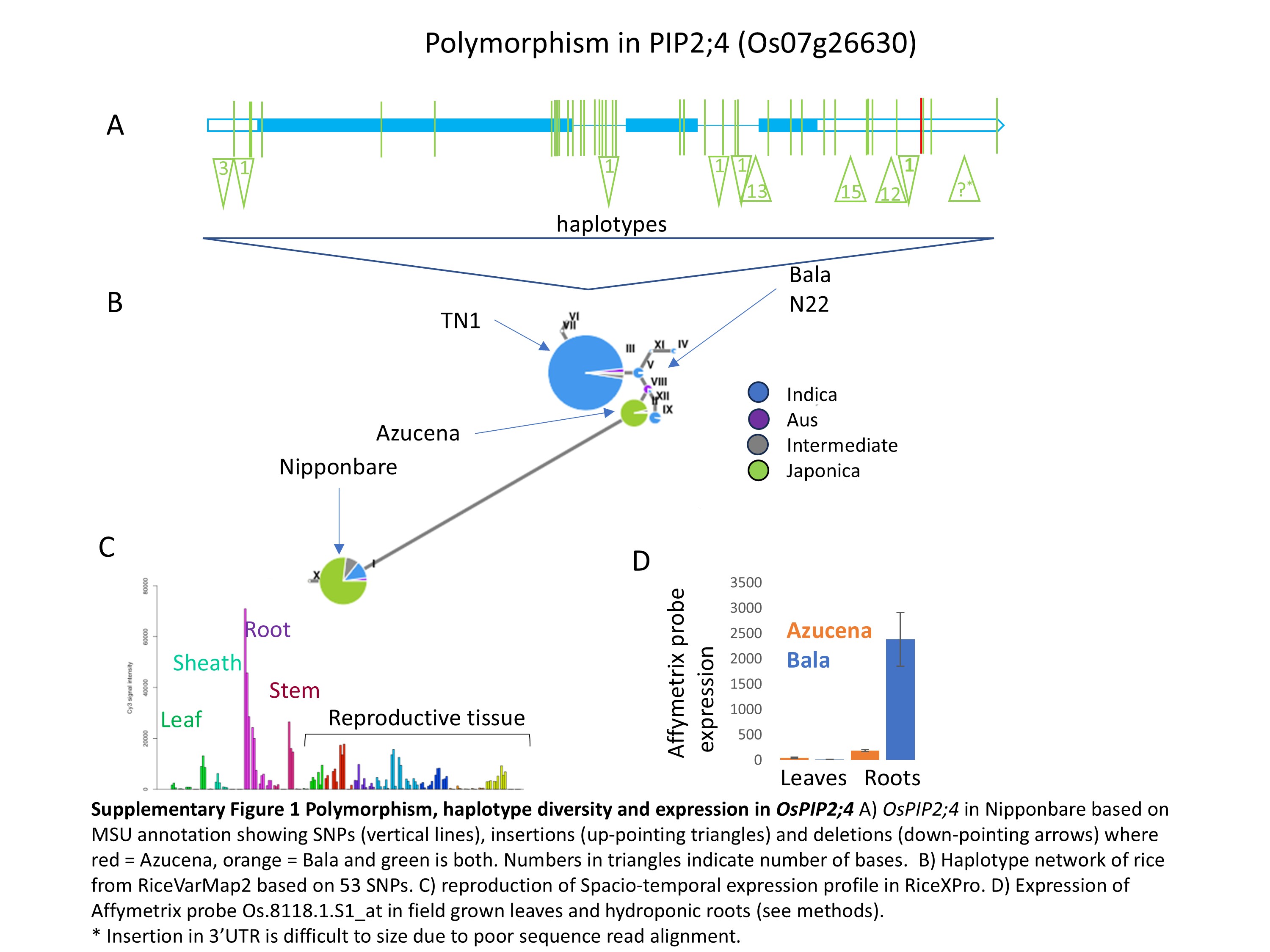

Supplement: Supplementary file 1 — Fig. S1. Polymorphism, haplotype diversity and expression in OsPIP2;4. (A) OsPIP2;4 in Nipponbare based on MSU annotation showing SNPs (vertical lines), insertions (up‐pointing triangles) and deletions (down‐pointing arrows) where red = Azucena, orange = Bala and green is both. Numbers in triangles indicate number of bases. (B) Haplotype network of rice from RiceVarMap2 based on 53 SNPs. (C) reproduction of spatio‐temporal expression profile in RiceXPro. (D) Expression of Affymetrix probe Os.8118.1.S1_at in field grown leaves and hydroponic roots (see Methods). * Insertion in 3′UTR is difficult to size due to poor sequence read alignment. [file PLB-28-1602-s003.jpg]

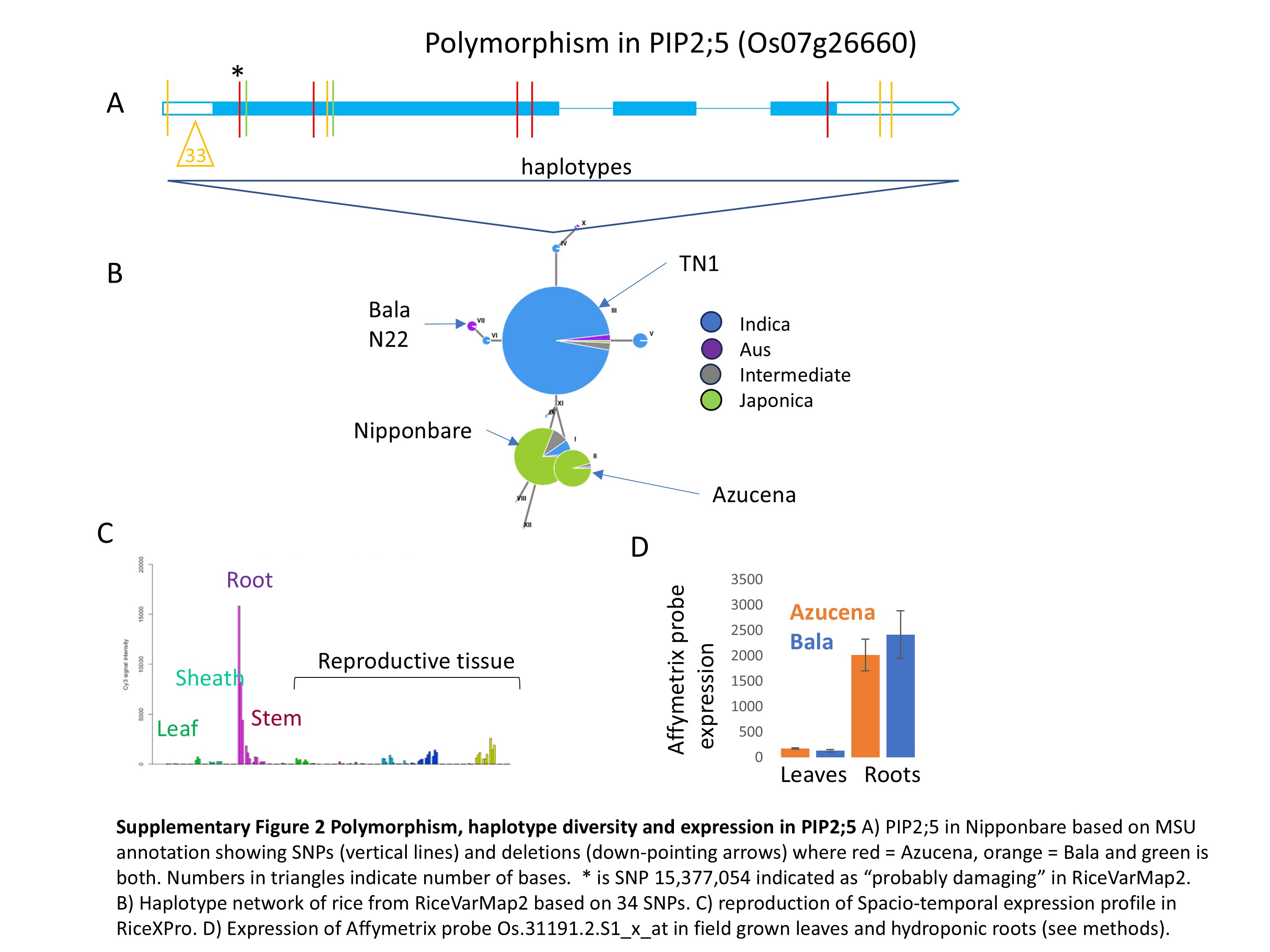

Supplement: Supplementary file 2 — Fig. S2. Polymorphism, haplotype diversity and expression in OsPIP2;5. (A) OsPIP2;5 in Nipponbare based on MSU annotation showing SNPs (vertical lines) and deletions (down‐pointing arrows) where red = Azucena, orange = Bala and green is both. Numbers in triangles indicate number of bases. * is SNP 15,377,054 indicated as ‘probably damaging’ in RiceVarMap2. (B) Haplotype network of rice from RiceVarMap2 based on 34 SNPs. (C) reproduction of spatio‐temporal expression profile in RiceXPro. (D) Expression of Affymetrix probe Os.31191.2.S1_x_at in field grown leaves and hydroponic roots (see Methods). [file PLB-28-1602-s002.jpg]

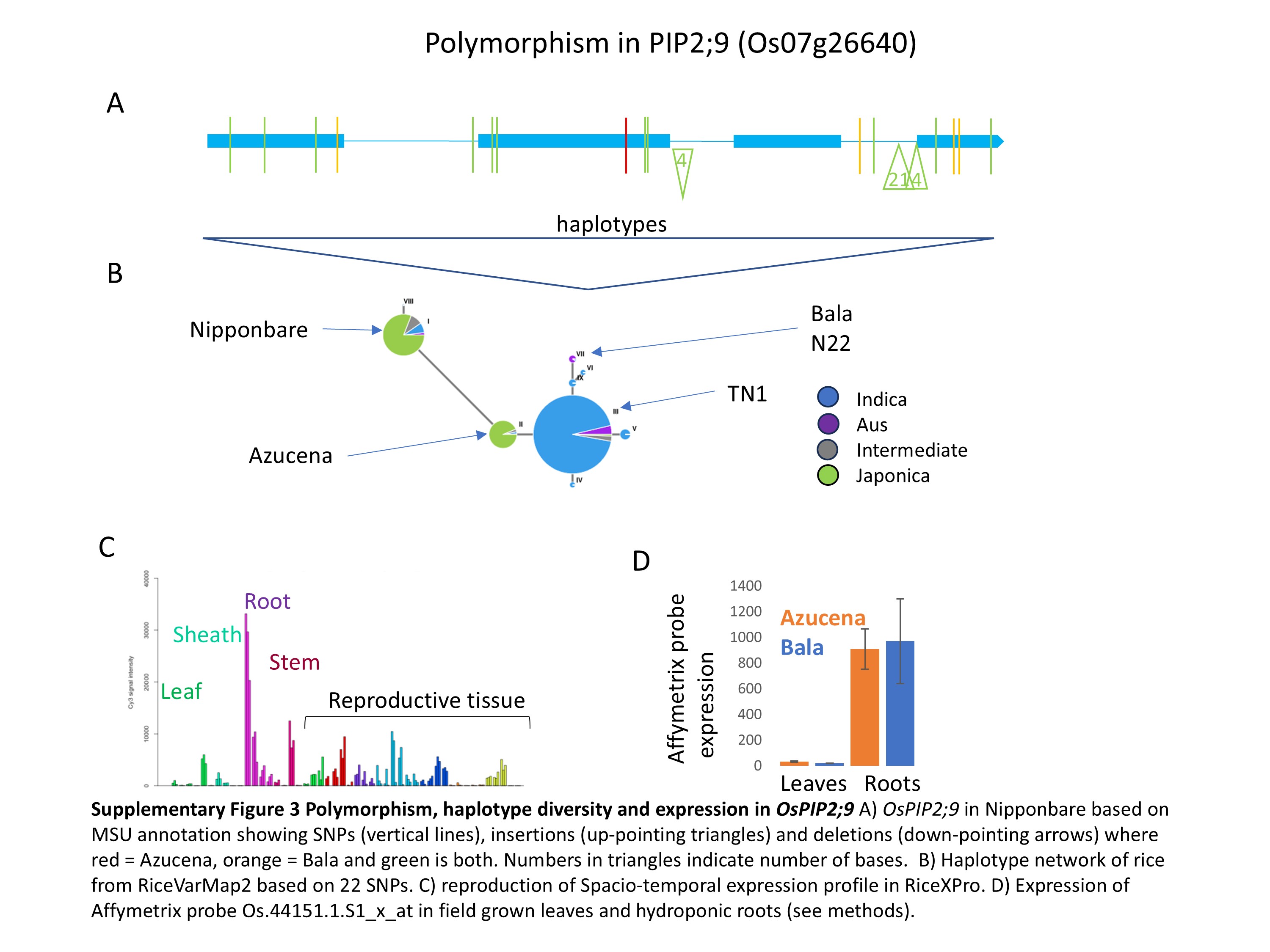

Supplement: Supplementary file 3 — Fig. S3. Polymorphism, haplotype diversity and expression in OsPIP2;9. (A) OsPIP2;9 in Nipponbare based on MSU annotation showing SNPs (vertical lines), insertions (up‐pointing triangles) and deletions (down‐pointing arrows) where red = Azucena, orange = Bala and green is both. Numbers in triangles indicate number of bases. (B) Haplotype network of rice from RiceVarMap2 based on 22 SNPs. (C) reproduction of spatio‐temporal expression profile in RiceXPro. (D) Expression of Affymetrix probe Os.44151.1.S1_x_at in field grown leaves and hydroponic roots (see Methods). [file PLB-28-1602-s001.jpg]
