# Supplementary material for: OsPIP2;1 impacts root hydraulic conductance and is a candidate gene for a drought avoidance QTL on rice chromosome 7
Source: Plant Biol (Stuttg). 2026 Jun 15;28(5):1602–13. doi: 10.1111/plb.70237 (PMC13358641; doi:10.1111/plb.70237)
Supplement: Supplementary file 4 — Data S1. Supplementary methods. [file PLB-28-1602-s004.docx]

**Supplementary Methods**

Selection of T1 plants with high and low expressions of *OsPIP2.1*.

T1 seeds from T0 plant TD4C8 1.2 were grown in hydroponics and the expression of *OsPIP2.1* relative to *OsRAC1* (actin) were assessed on RNA extracted from roots of individual plants (using methods described in the article including three technical replicates). From the results below, TD4C8 1.2/G was selected as a low expression line, and TD4C8 1.2/I was selected as high expression.

The same approach was applied to seven T1 plants from T0 plant TD3C8 5.1. From the results below, TD3C8 5.1/E was selected as a low expression line, and TD3C8 5.1/I was selected as high expression

Selected T1 plants were grown for production of T2 seeds for subsequent experiments. Note differences in expression in the T2 progeny of the selected plants was subsequently confirmed in biologically replicated experiments shown in figure 4.
